# Duplication of the Alar Ligaments: A Case Report

**DOI:** 10.7759/cureus.2893

**Published:** 2018-06-29

**Authors:** Asad Rizvi, Joe Iwanaga, Rod J Oskouian, Marios Loukas, R. Shane Tubbs

**Affiliations:** 1 St. Georges University School of Medicine, St. Georges, GRD; 2 Seattle Science Foundation, Seattle, USA; 3 Neurosurgery, Swedish Neuroscience Institute, Seattle, USA; 4 Anatomical Sciences, St. George's University, St. George's, GRD; 5 Neurosurgery, Seattle Science Foundation, Seattle, USA

**Keywords:** alar ligament, duplication, craniocervical junction, variant, transverse occipital ligament, anatomy

## Abstract

The alar ligament is one of the two strongest ligaments stabilizing the craniocervical junction. The literature describes many variations of the attachment, insertion, shape, and orientation of the alar ligament and an understanding of these variations is vital as they can lead to altered biomechanics or misinterpretation on imaging. Herein, we report, to our knowledge, the first case of duplication of the alar ligaments and discuss the anatomical variations present in the literature.

## Introduction

The craniocervical junction is composed of the atlantooccipital and the atlantoaxial joints. Several ligaments stabilize these joints, namely the transverse, alar, transverse occipital, accessory, lateral atlantooccipital, and apical ligaments [1]. The transverse and alar ligaments are the two strongest ligaments stabilizing the craniocervical junction with approximately 400 N and 200 N necessary until failure, respectively [1-2].

The alar ligaments are fibrous cords that attach to the dens bilaterally and insert on the base of the skull. They function to limit axial rotation and lateral bending on the contralateral side, and flexion secondarily [1-2]. These functions are a result of the specific attachment, insertion, and orientation of the fibers of the alar ligaments and variations in these can lead to altered biomechanics. Additionally, knowledge of the anatomical variants is essential from a surgical standpoint [3].

Many anatomical variations of the alar ligaments have been described [4-17], but as far we are aware, there is no report of the duplication of the alar ligaments. Herein, we present the first case of duplication of the alar ligaments in a cadaveric specimen and discuss some anatomical variations of the alar ligaments present in the literature.

## Case presentation

**Figure 1:**
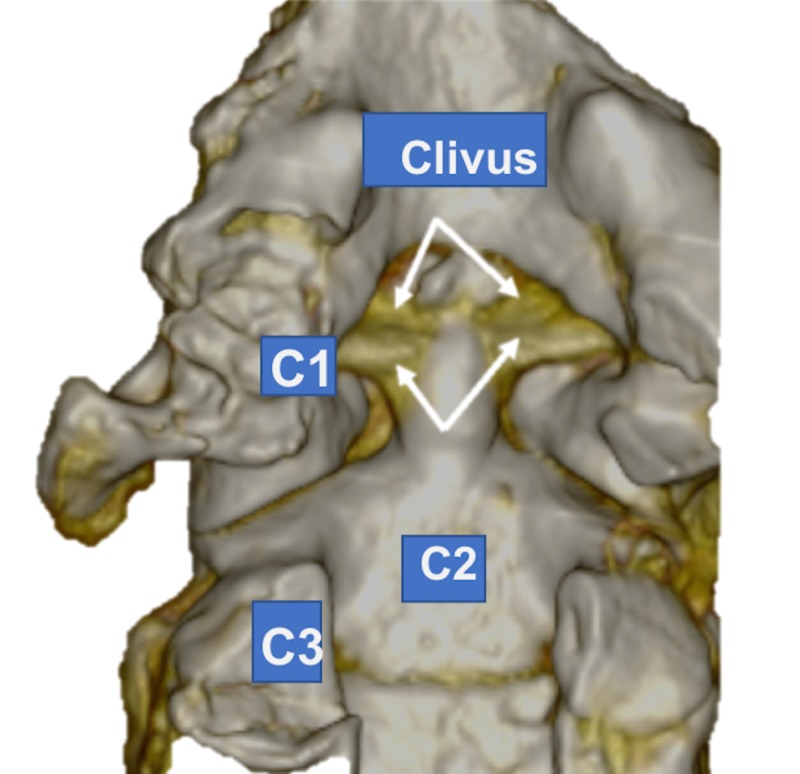
3D computed tomography (CT) reconstruction of the duplicated alar ligaments (arrows) as seen in the case reported here

During the routine dissection of the craniocervical junction in an adult, fresh frozen, male cadaver, an unusual ligamentous complex was found. From a posterior approach, the dura mater overlying the C2 vertebra was removed and the tectorial membrane removed. Once the cruciate ligament was identified, it was removed to visualize the posterior aspect of the dens. At this point, the alar ligamentous complex was found to be more robust than normal. With additional dissection, it became clearer that the alar ligament on the left and right sides was actually duplicated in, more or less, the coronal plane. But with the duplicated parts being slightly anterosuperior to the normally positioned ligaments. The specimen underwent computed tomography (CT) imaging (Figure 1). The duplicated parts were more or less of the same caliber of the normally positioned alar ligaments. Both the normally positioned alar ligaments and the duplicated ligaments arose from the lateral aspect of the dens, the duplicated ones slightly more anterior and superior, and these all attached along the medial edge of the occipital condyle. No transverse occipital ligament was identified. With the rotation of the craniocervical junction, both ligaments restricted rotation but not grossly, more so than would be seen with single left and right alar ligaments. No other anatomical variations or pathology of the region dissected was noted.

## Discussion

Attachment onto the dens

The attachment site of the alar ligaments onto the dens has been variously reported as on the lateral margin of the posterior surface of the upper one-third of the dens [4], the dorsolateral surface of the tip of the dens [5], the apex of the dens [6], or on the lateral aspect of the apex of the dens (64% of the cadavers with mean age at death of 78.9 years) [7]. Considering this variation, Sardi et al. [8] designed a study to explore the attachments of the alar ligaments (observed from three views) on the dens of 22 sides of 11 cadaveric specimens. When observing from a posterior view, 16 (72.7%) alar ligaments attached onto the posterolateral aspect and six (27.3%) passed over the tip of the dens. When observing from a superior perspective, 14 ligaments covered the posterior two-thirds of the dens, and two ligaments covered the dens completely. Importantly, all ligaments were inserted posterolaterally on the dens when observing from above, and leading to an almost horizontal orientation of the fibers toward the occipital condyles. This orientation allows the alar ligaments to limit flexion and anterior translation [8].

Insertion and shape

There are some reports of the insertion site of the alar ligament onto the medial aspect of the occipital condyle [5-6,9-11], and some on the lateral walls of the foramen magnum [4]. One author [11] reported insertions on the medial aspect of the occipital condyle in 10 out of 11 (90.9%) cadavers studied and suggested that the insertion on the foramen magnum is an anatomical variant. The literature describes the shape of the ligaments as tubular [12], elliptical, or rectangular [4]; and the cross-sectional shape as ovoid, wing-like, or round [13].

Orientation of fibers

Variations of the directions of the fibers of the alar ligaments have also been reported. One study [14] on 19 specimens described a craniocaudal orientation in nine specimens (47.4%), horizontal in six (31.6%), and caudocranial in four (21.0%). However, others [11] have observed a higher frequency of the horizontal orientation with one author reporting this orientation in seven out of 11 specimens (63.6 %) [15]. The horizontal orientation is “more accurate” compared to other orientations as it allows the alar ligaments to limit rotational movements, while a vertical orientation would make this “inefficient” [11].

Attachment to the atlas

Attachment of the alar ligaments to the atlas has been reported as a connection between the dens and lateral mass of the atlas with fibers oriented “obliquely craniocaudally” [14]. However, other studies have not found this connection [11-13,15]. The authors of a study designed to investigate this atlantal connection note two possible explanations for this discrepancy: 1) the atlantal portion might exist in a subgroup of the population that were included in previous studies and might not usually be present in a larger sample of the population, and 2) the connections between the dens and the atlas are not part of the alar ligaments [15].

Transverse occipital ligament

A band of fibers previously described as a portion of fibers that pass above or behind the dens without any attachment to it and connecting with the contralateral alar ligament [16], is now identified as the transverse occipital ligament [15]. Studies report frequencies of this ligament in the examined specimens as 8.3% [17], 77.8% [18], and 44% [19]. Our case does not represent a transverse occipital ligament as the duplicated alar ligament fibers were anterior to the normally positioned alar ligaments, was represented bilaterally and was of a much larger caliber. The transverse occipital ligament extends across the foramen magnum attaching to the occipital condyles and inserts posterior to the lateral attachments of the alar ligaments [18]. Three categories have been described based on the attachment to the atlas and dens: Type I with connections to the alar ligaments on both sides and to the dens; Type II with connections to the alar ligaments on both sides, but none to the dens; and Type III with no connections to the alar ligament or the dens [19].

The transverse occipital ligament is located superior to the transverse ligament and posterosuperior to the alar ligaments [18]. Due to its location and similar morphology, the transverse occipital ligament functions with the alar ligament to limit axial rotation, lateral bending, and flexion [18]. Finally, an accessory band of tissue of the alar ligament oriented vertically toward the occiput has been previously described [16].

## Conclusions

The alar ligament is a thick fibrous cord that attaches to the lateral aspect of the dens and inserts on the medial aspect of the occipital condyle. Here, we report, to our knowledge, the first case of duplication of the alar ligaments and discuss other anatomical variations of the alar ligament concerning its attachment, insertion, and orientation. Knowledge of such variants is essential for clinicians operating or viewing imaging of the craniocervical junction.
